# Fermentation in breadmaking: enhancing digestibility and nutritional value for gastrointestinal health

**DOI:** 10.3389/fnut.2026.1777596

**Published:** 2026-05-21

**Authors:** Léa Ribet, Robin Dessalles, Héliciane Clement, Laura Nyhan, Nathália Trunckle Baptista, Yaqin Wang, Elke K. Arendt, Mickaël Durand-Dubief

**Affiliations:** 1 Lesaffre Institute of Science & Technology, Lesaffre, Marcq-en-Barœul, France; 2 Nutrition & Health, Robin Dessalles EI, Lyon, France; 3 School of Food and Nutritional Sciences, University College Cork, Cork, Ireland; 4 Department of Food and Nutrition, University of Helsinki, Helsinki, Finland; 5 APC Microbiome Institute, University College Cork, Cork, Ireland

**Keywords:** cereals, dough, fermentation, FODMAPs, gastrointestinal health, gluten, lactic acid bacteria, yeast

## Abstract

Fermentation is a traditional food process that has been used for ages, notably contributing to the production of bread, one of the main staple foods. The aim of this review is to explore how fermentation may be leveraged to enhance the digestibility and nutritional value of bread, addressing potential gastrointestinal disorders related to its consumption. Emerging gastrointestinal disorders such as non-celiac gluten or wheat sensitivity and irritable bowel syndrome (IBS) are discussed, highlighting the complex and often poorly understood, nature of these conditions involving both physiological and psychological aspects. Yeasts and lactic acid bacteria used in the bread making process can positively influence bread characteristics through the enhancement of mineral bioaccessibility or the reduction of triggering components like gluten and FODMAPs, especially when selecting microorganisms with targeted properties. However, the characterization and selection of these microorganisms require further standardization, and the clinical implications need to be firmly established as the strength of the evidence remains insufficient. The review also discusses the potential for fermentation to generate beneficial metabolites, such as exopolysaccharides (EPS) and short-chain fatty acids (SCFA). While these compounds show promise, their actual content, stability, and bioavailability in baked bread require further investigation. Overall, fermentation appears as a versatile transformative tool for the creation of digestible and nutritionally enhanced bread. Yet well-designed human studies are needed to substantiate the clinical benefits and better define the conditions where modification through fermentation may provide gastrointestinal health advantages.

## Graphical Abstract

**Figure:**
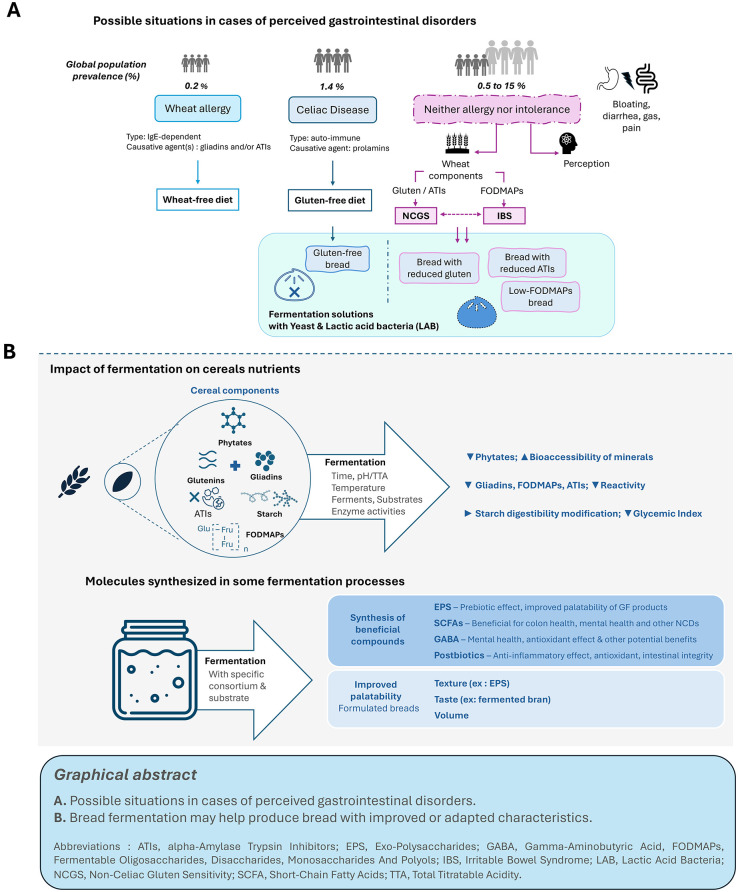

## Introduction

Bread stands out as one of the main staple foods consumed worldwide and is a significant contributor to nutritional intake (1–3), especially in European countries (1, 4–7) and in the United States (8). Over the past decades, concerns have arisen regarding the health consequences of consuming wheat bread, and more specifically gluten, a complex of storage proteins conferring the dough its viscoelastic properties. These concerns have spread widely, especially in Western societies, through the press and the internet. This has resulted in increased popularity of the gluten-free (GF) diet, which has become a perceived healthy option (e.g., promoted for weight loss) for a significant proportion of the population, despite the absence of scientific evidence supporting these claims (9–11). Nonetheless, in most cases, gluten is not the cause of gastrointestinal trouble, and other triggering factors appear to be involved, such as fermentable carbohydrates known as FODMAPs (for Fermentable Oligosaccharides, Disaccharides, Monosaccharides And Polyols), poorly absorbed in the human small intestine, thus potentially inducing gastrointestinal symptoms in sensitive individuals. These compounds are typically involved in a condition called the irritable bowel syndrome (IBS) (12, 13).

Fermentation is a traditional food process that has been widely used throughout human history for a variety of purposes, from making food suitable for human consumption, to preservation, enhanced palatability and improved taste (14, 15). Fermented foods are defined as “*foods made through desired microbial growth and enzymatic conversions of food components*.” (16)

It is already known that fermentation can be used to improve the nutritional value of bread, and generate beneficial metabolites (17). More recently, bread made using specific fermentation processes has been shown to improve aspects of gastrointestinal or metabolic health, notably through its ability to degrade components potentially source of gastrointestinal discomfort (18).

In this context, the present review will (1) cover current knowledge regarding emerging gastrointestinal disorders related to bread consumption (2) explore how fermentation can be leveraged to enhance the digestibility and nutritional value of bread, and (3) discuss the potential of fermentation for reducing triggering components and generating beneficial metabolites.

## Nutritional implications of emerging gastrointestinal disorders

Complaints of gastrointestinal symptoms following wheat or gluten ingestion have dramatically increased in the general population (12, 13).

The scientific evidence is unequivocal regarding the health improvements following gluten exclusion from diet in the case of celiac disease (CD), an autoimmune disease triggered by gluten from diet in genetically predisposed individuals (19). Similarly, wheat exclusion is the recommended treatment in case of wheat allergy (WA), an immunoglobulin E-mediated hypersensitive reaction, most encountered in children, and caused by the presence of gliadins or by a group of enzyme inhibitors present in wheat called α-Amylase-Trypsin Inhibitors (ATIs). The diagnosis of both these conditions relies on well-defined biological markers (10). The world prevalence of both conditions is estimated to be 1.4% for celiac disease (20, 21), and 0.2% for wheat allergy (22).

Concomitantly, an increasing proportion of individuals still report gastrointestinal and extra-intestinal symptoms related to wheat or gluten, though the diagnoses of celiac disease and wheat allergy have been ruled out. Usually, the symptoms seem to improve under a gluten-free diet, hence the denomination of Non-Celiac Gluten Sensitivity (NCGS) or Non-Celiac Wheat Sensitivity (NCWS) for this cluster of symptoms (23). This led to the following definition: “*Non-Celiac Gluten Sensitivity (NCGS) is a syndrome characterized by intestinal and extra-intestinal symptoms related to the ingestion of gluten-containing food, in subjects that are not affected by either celiac disease (CD) or wheat allergy (WA)*.” (24)

Despite this definition, establishing a precise clinical picture is challenging, as the clinical manifestations of NCGS are heterogeneous, and the symptoms overlap with a range of other conditions, including IBS, a common functional gastrointestinal disorder characterized by the presence of recurrent abdominal pain with changes in stools frequency and/or appearance (24). This may in part explains the significant variation in NCGS prevalence reported throughout the literature, ranging between 0.49 and 14.9% depending on studies and geographical areas (23), with an estimated pooled prevalence of 10% based on data from 16 countries (25).

Due to the lack of validated biomarkers, NCGS diagnosis relies primarily on self-report. Capannolo et al. demonstrated the poor predictive value of self-diagnosis of NCGS in a study including 392 individuals reporting gluten-related symptoms. Indeed, after exclusion of patients with CD or WA, a gluten-free diet resolved symptoms in only 7% of these individuals, while no improvement was seen in 86% of cases. Thus, most of these individuals could not be considered NCGS, and their actual condition remained undetermined (26). In several studies, patients with self-reported NCGS had alleviations of their symptoms under a low-FODMAPs diet (11), or seemed to have a higher symptoms score after a fructan challenge than after a gluten challenge (23, 27), suggesting that FODMAPs may be the cause of their symptoms rather than gluten. A review of 10 randomized controlled trials reported that only 16% of patients with self-reported NCGS had gluten-specific symptoms following a double-blind, placebo-controlled, gluten challenge. In addition, the authors highlighted that in 40% of the patients, a nocebo response occurred, i.e., these patients experienced similar or worsened symptoms during the placebo challenge compared to the gluten challenge (28). In a study of adolescents self-reporting gastrointestinal symptoms, Crawley et al. showed that an open run-in period under a gluten-free diet improved symptoms in 33 out of 54 subjects of the subjects. However, in the subsequent blinded randomized cross-over challenge, no significant differences in symptoms could be reported between gluten or placebo groups. The authors mentioned the presence of a strong nocebo effect (29). de Graaf et al. aimed at assessing the impact of negative expectations on gastrointestinal symptoms score in 84 adults given gluten or placebo, in a randomized, double-blind, placebo-controlled design. After a 1-week run-in period demonstrating the participants responded positively under a gluten-free diet, they were divided into four groups: patients expected to ingest gluten, and actually received gluten-containing bread (E+G+); patients expected to ingest gluten, but actually received gluten-free bread (E+G–); patients did not expect to ingest gluten, but actually received gluten-containing bread (E–G+) and; patients did not expect to ingest gluten, and actually received gluten-free bread (E–G–). The authors reported that the groups expecting to receive gluten reported significantly more gastrointestinal symptoms compared to those not expecting it, whether they actually received gluten or not, confirming an important nocebo effect (30).

Another randomized, single-blind, crossover study could not identify differences in extra-intestinal symptoms or gastrointestinal symptoms in subjects receiving gluten or placebo, whether they were healthy or diagnosed with NCGS. However, NCGS patients had significantly higher negative affect scores and lower positive affect scores compared to healthy controls, irrespective of the attributed group (gluten or placebo). The authors concluded on a lack of gluten-specificity regarding the symptoms, and on the presence of baseline negative psychological affects in NCGS (31).

Additionally, one study showed that the poor predictive value of self-perception of gluten sensitivity also occurs in IBS patients. Indeed, these individuals reacted similarly to gluten, wheat, or placebo in a randomized, double-blind, sham-controlled crossover study. Of note, 1 month after study disclosure, most participants still believed the gluten-free diet to be effective (32). This nocebo effect in individuals diagnosed with IBS is in line with results from previous works (33).

Overall, the poor predictive value of self-diagnosis, the absence of discriminating clinical symptoms, and the presence of a strong nocebo effect suggest that most individuals reporting gluten-related symptoms may inappropriately adopt a gluten-free diet (34).

Indeed, gluten avoidance has become increasingly common in Western countries. In France, for example, the National Dietary Intake survey (INCA study) indicated that during the 2014–2015 period, 11.4% of adults avoided gluten due to intolerance or allergy (6). The NUTRINET Santé survey reported that 10.31% of the participants avoided gluten, of which 1.65% totally (35). Meanwhile, according to the National Health and Nutrition Examination Survey (NHANES) in the United States, the prevalence of adults on a gluten-free diet without a diagnosis of CD increased from 0.5% in 2009–2010 to 1.7% in 2013–2014, while the prevalence of undiagnosed CD decreased during this time (36, 37). In the United Kingdom, it has been reported that 3.7% of the general population was avoiding gluten (38).

Concomitantly, the global market of gluten-free foods has shown spectacular growth, led by North America and Europe (37, 39), with a value estimated at $7.75 billion in 2024 expecting to reach $13.67 billion by 2030 (40).

Despite the widespread perception that gluten-free diets are beneficial for general health and wellbeing, research indicates that many gluten-free products seem to have a lower nutrient density compared to their gluten-containing counterparts. In a comprehensive review of 46 studies reporting the nutritional content of gluten-free foods worldwide, finding showed that gluten-free containing products contained less protein, more fat, variable fiber content, and were rarely fortified with micronutrients (41, 42). Furthermore, a meta-analysis of 18 studies evaluating 132 samples of gluten-free bread from all over the world highlighted that these products generally have a high glycemic index (GI) (43). These observations have been made in a variety of countries, including Brazil (44–46), Chile (47), the UK (205, 206), Spain (48, 207), Norway (208), Canada (209), Australia (210), or India (49).

Despite the diversity of individual physiological and social circumstances, some people may choose to consume gluten-free foods for well-founded reasons, and significant progress has been made over the past decade in providing these individuals with sensory-appealing and nutritionally-adequate gluten-free products (50). Research has identified promising ingredient combinations that may improve the quality of GF products. Indeed, most of the gluten-free products on the market appear to be obtained from refined corn or rice flours, which may impact products quality (51). Studies have shown better results on both textural and nutritional properties when selecting other cereal flours, such as sorghum, millet, teff, buckwheat, quinoa, or amaranth (52). Also, the use of dietary fiber ingredients may be useful in this respect, as reported by studies adding inulin, oat ß-glucan, psyllium or resistant starch (RS) (53). Hydrocolloids such as xanthan gum, guar gum, carboxymethyl cellulose or carrageenan may also both improve gluten-free bakery products characteristics (54) and GI (55). Finally, micronutrient content has been improved in gluten-free products also by using alternative flours for example, buckwheat, flaxseed, or amaranth (52).

Apart from gluten, there are many causative agents potentially generating gastrointestinal distress in patients with a similar clinical picture than NCGS. These include FODMAPs or wheat ATIs (12). A viable alternative strategy to gluten-free foods could be a more specific approach targeting triggering components, while proposing foods with high-nutrient and low-energy density. In this respect, fermentation may be a useful processing tool. The next sections will therefore explore how ferments, and notably those involved in the breadmaking process, could be of use in this respect.

## Using fermentation for enhancing foods

Breadmaking is a complex technological process involving multiple steps, from ingredient selection and dough mixing to fermentation, proofing, and baking. This complexity offers potential to modulate the nutritional properties of bread and possibly generate diverse health-related outcomes depending on the type of flour used (refined vs. whole grain, legume flours, etc), fermentation strategy (sourdough, straight dough fermentation using baker's yeast, or yeast-based preferments, e.g., poolish, biga, sponge process) and processing conditions applied (56).

### Ferments in bread-making, characteristics, and diversity

Historically, dough leavening was done using spontaneous fermentation, consisting in the activation of naturally occurring microorganisms and enzymes in milled grains by adding water. Eventually, the use of sourdough, i.e., starting the fermentation with material from the previous fermentation process became more systematic, improving process consistency and product quality (15).

Fermentation for breadmaking relies on two key microbial groups: yeasts and lactic acid bacteria (LAB) (57). These microorganisms play distinct yet complementary roles in conventional dough and sourdough systems.

Yeasts /are the main actors of fermentation in processes such as brewing, baking, wine making (58). The term “yeast” is often taken as a synonym for *Saccharomyces cerevisiae*, the so-called baker's yeast, but this species is not the only yeast used for breadmaking, especially in sourdough (59–61). *Saccharomyces cerevisiae* (baker's yeast) has become the predominant leavening agent since the 19th century, offering faster fermentation than sourdough, reducing working time for bakers, and producing bread with lighter crumb and milder taste (62, 63).

The diversity among *S. cerevisiae* strains offers significant advantages in breadmaking (64, 65). In dough, yeasts convert fermentable sugars into carbon dioxide and ethanol, causing the dough to rise (66, 67). Yeast metabolism significantly influences bread quality through CO_2_ production, stress tolerance, and formation of compounds like glycerol and formation of compounds that affect dough properties and final bread characteristics (68, 69). Fermenting yeasts also produce a range of aromatic compounds through amino acid metabolism (60, 61) allowing for tailored selection to achieve desired sensory attributes.

LAB, the second key microbial group, are gram-positive, acid-tolerant bacteria used various fermentation processes, yielding a number of products such as cheese, yogurt, kefir, sauerkraut, or sourdough bread (56). They produce specific molecules such as aromas and flavor precursors, bioactive or antimicrobial compounds. While LAB play a minor role in conventional dough fermentation, they are essential in sourdough systems where they are predominant and significantly influence dough properties and bread quality.

The concept of maintaining a stable spontaneous microbial community in an active state through regular backslopping, commonly known as “traditional sourdough” or “type I sourdough,” remains in use today, primarily in artisanal bakeries. Hammes et al. (70) described the production of two other types of sourdough called “type II” and “type III.” Type II sourdoughs are mostly in liquid form and used at industrial scale and in German bakeries for their acidifying rather than leavening effect. Type III sourdoughs are type II sourdoughs which have undergone drying after fermentation, which makes it more convenient for industrial bakeries and the production of standardized end-products (71).

About 80 different LAB species have been identified in sourdough [for review, (72)] mostly comprising various reclassified *Lactobacillus* genera such as *Lactiplantibacillus* and *Fructilactobacillus*, alongside *Weissella, Leuconostoc, Pediococcus*, and *Lactococcus* (59, 73). The broad diversity of LAB associated with sourdough fermentation leads to considerable variation in growth parameters and stress resistance. Sourdoughs with different fermentation parameters will favor the growth of distinct microbial communities. Traditional spontaneous sourdoughs, called “type I sourdoughs,” are characterized by relatively short fermentation times not exceeding 24 h and thus, tend to favor microorganisms with rapid growth on flour-based media (74) such as *Fructilactobacillus sanfranciscensis* (75). In contrast, type II sourdoughs, which are typically liquid sourdough starters with extended fermentation times at high temperatures, favor the development of acid-tolerant LAB species (76, 77). The optimal pH for LAB in sourdough is generally between 5 and 6, which corresponds to the pH of sourdough at the start of fermentation when 5%−20% ripe sourdough is added during back slopping (78, 79).

Yeasts isolated from sourdough include over 20 different species with the most commonly found *Saccharomyces cerevisiae, Maudiozyma humilis, Pichia kudriavzevii, Maudiozyma exigua, Torulaspora delbrueckii* and *Wickerhamomyces anomalus* (59). This diversity can be explained either by the raw material origin, as the genera *Candida, Cryptococcus, Maudiozyma, Pichia, Rhodotorula, Torulaspora, Trichosporon, Sporobolomyces*, and *Saccharomyces* were also isolated from grains, or by the process parameters of sourdough propagation, such as hydration, temperature, time between backsloppings, etc. (80).

In traditional sourdoughs, yeasts and LAB interact with each other during fermentation, serving complementary functions. Yeasts (primarily *S. cerevisiae* in conventional bread) generate carbon dioxide for leavening while also producing aromatic compounds. LAB produce organic acids that acidify the dough, contributing to flavor development and preservation properties. Some yeasts like *Maudiozyma humilis* or *Kazachstania exigua*, cannot use maltose as carbon source, but sucrose or glucose. Conversely, *F. sanfranciscensis* catabolizes maltose, preventing therefore competition between these species for fermentation substrates. Moreover, *F. sanfranciscensis* fermentation releases glucose into the medium, which the yeasts can then use. In parallel, fructose, produced by the hydrolysis of sucrose under the action of yeast invertase, serves as a secondary electron acceptor for LAB, increasing the production of acetic acid (81). Hence, there can be beneficial interactions between LAB and yeast in traditional sourdough. As a result, the CO_2_ formed by yeast, and to a lesser extent by bacteria, is produced in greater quantities when these two organisms are combined (82).

The combination of LAB and yeast creates synergistic effects on bread quality. Yeast produces aromatic compounds from amino acids, a process enhanced by the proteolytic activities of lactic acid bacteria and endogenous enzymes in the flour, which are activated by the pH drop. Additionally, this acidic environment induces other changes in the dough matrix, including amylase inactivation, gluten and starch structures alterations and increased fiber solubilization (83). All these changes, regarded as sourdough performances, significantly influence the characteristics of leavened baked goods. Sourdough fermentation improves bread flavor (61) and texture (84), enhancing its palatability while extending shelf-life by delaying both staling (85) and mold spoilage development (86).

Finally, the interaction of LAB and yeasts may also bring benefits beyond bread texture and flavor; it may for instance improve nutritional density, enhance fiber or carbohydrate content, or degrade anti-nutritional or compounds source of gastrointestinal trouble. A summary of studies using ferments for these applications is provided in Table 1. These points are detailed in the next sections.

**Table 1:** Ferments impacting nutrient content of breads.

Functionality	Effect on nutritional characteristics of bread	Strain/Species/specificities	Type of evidence	References
Phytic acid reduction (i.e., improved mineral bioaccessibility of Fe, Mg, Zn, P)	Phytase activation via acidification: optimal environment for endogenous phytase activity (pH 4.3-5.5)	*Saccharomyces cerevisiae* *Lactobacillus brevis*	*in vitro*	(93)
		*Saccharomyces cerevisiae* with organic acids (optimal pH 4,5)	*in vitro*	(94)
Increase of Resistant starch (RS)	Increased of resistant starch content	*Lactobacillus brevis* ELB99 *Lactiplantibacillus plantarum* ELB75 *Saccharomyces cerevisiae* TGM55	*in vitro*	(102)
		Long sourdough fermentation	*in vitro*	(103)
	Starch retrogradation during staling using sourdough	Not specified	*in vitro*	(106)
		*Lactobacillus fermentum*	*in vitro*	(107)
Reduction of gluten	Degradation of 33-mer epitope and other gliadin fragments	*L. alimentarius* 15M, *L. brevis* 14G, *L. sanfranciscensis* 7A, and *L.hilgardii* 51B	*in vitro*	(133)
		*Lactobacillus casei* *Leuconostoc mesenteroides* *Lactobacillus plantarum*	*in vitro*	(135)
		*Lactobacillus alimentarius 15M* *Lactobacillus brevis 14G* *Lactobacillus sanfranciscensis 7A* *Lactobacillus hilgardii 51B*	*in vitro*	(134)
		*Bacillus* spp *especially GS 188*		(136)
		Mix of *L. casei* LC130, *L. paracasei* LPC100 and *S. thermophilus* ST250		(137)
	Reduction of up to 78% of α-gliadins	*Wickerhamomyces anomalus* yeast	*in vitro*	(143)
	Sourdough fermentation mitigates risk of cross-contamination of gluten-free (GF) products	*L. sanfranciscensis LS40 and LS41, and L. plantarum CF1 with GF flours* *L. sanfranciscensis and Candida humilis with GF flour* *Lactobacillus amylovorus with quinoa flour*	Review	(145) WZ
	Reduction of gluten to <20 ppm (gluten-free threshold) in wheat	*L. sanfranciscensis* strains (7A, LS3, LS10, LS19, LS23, LS38, and LS47) and/or *Lactobacillus alimentarius* 15M, *Lactobacillus brevis* 14G, and *Lactobacillus hilgardii* 51B and fungal proteases (from *Aspergillus oryzae* and *A. niger*)	*in vitro*	(146, 147)
Reduction of FODMAPs	Reduction of 69% fructans and raffinose reduction but increase of mannitol (550%)	Sourdough (composition not specified; commercial starter)	*in vitro*	(18)
	Reduction of FODMAP by >65% compared to whole-wheat flour	Baker's yeast *Saccharomyces cerevisiae*	*in vitro*	(153)
	Reduction of fructans to a final level of 0.3% dry matter attributed to high invertase activity in whole wheat breads	Baker's yeast *Saccharomyces cerevisiae* (28 strains)	*in vitro*	(155)
	Reduction of >90% of fructans in whole wheat breads	*Kluyveromyces marxianus* (30 strains)	*in vitro*	(156)
	Reduction of fructans and fructose in whole-wheat bread	*Lachancea fermentati* FST 5.1, *Cyberlindnera fabianii* NTCyb	*in vitro*	(157)
	Reduction of fructans in whole-wheat bread	*Candida glabrata* *Maudiozyma humilis*	*in vitro*	(158)
		*Kazachstania exigua* *Kluyveromyces marxianus* *Pichia kudriavzevii* *Torulaspora delbrueckii* *Torulaspora pretoriensis* *Wickerhamomyces anomalus* *Candida lambica, Hanseniaspora uvarum, Saccharomyces cerevisiae* (2 strains) *Torulaspora delbrueckii*		
	Reduction of FODMAPs by more than 90%	Baker's yeast *Saccharomyces cerevisiae*	*in vitro*	(161)
	Specific cultures as starters in sourdough reduced fructans content by >92%	*Levilactobacillus brevis* *Weissella minor* *Lactiplantibacillus plantarum* *Leuconostoc citreum, Limosilactobacillus fermentum* *Companilactobacillus farciminis*	*in vitro*	(162)
	Fructans reduction of less than 50% in a straight dough bread	Baker's yeast *Saccharomyces cerevisiae*	*in vitro*	(161)
	Conventional sourdough fermentation reduced fructans in bread by 65%−70%	Sourdough (composition not specified)		
	*L. crispatus* sourdough reduced fructans by 90%	*Lactobacillus crispatus* DSM29598		
	Reduction of fructans up to 81% in light flour wheat bread with sourdough addition	Sourdough: *Lactiplantibacillus plantarum* 20174 and Baker's yeast *Saccharomyces cerevisia*e	*in vitro*	(160)
Reduction of ATIs	All isolates exhibited the potential to degrade ATIs to a high degree	87 LAB isolates and collection strains	*in vitro*	(164)
	ATI degradation capacities ranged from 52.3%−85.0% by HPLC and 22.2%−70.2% by ELISA, with *Lacticaseibacillus paracasei* Lpa4	*Lacticaseibacillus paracasei* Lpa4		
	When pH of sourdough is below 4.0, ATI tetramers were degraded compared to yeast fermentation.	Not specified (Sourdough)	*in vitro*	(165)
	Tetrameric ATI structures were unraveled in yeasted and sourdough breads vs. baking flour, but the overall reduction in ATIs to their monomeric form was higher in the sourdough bread group	Not specified (Sourdough)	Randomized, double-blind, controlled clinical study	(167)
	Longer sourdough fermentation time (12 h) caused up to 41% reductions in ATIs in the resulting sourdoughs	Not specified (commercial sourdough starter)	*in vitro*	(18)

The table presents the main species of lactic acid bacteria and yeasts playing a role on specific bread components.

LAB, Lactic Acid Bacteria; spp., species (plural); ATI, α-Amylase-Trypsin Inhibitors; dm, dry matter; FODMAPs, Fermentable Oligosaccharides, Disaccharides, Monosaccharides and Polyols.

### Using fermentation for enhancing nutritional quality

While the nutritional profile of bread is primarily determined by ingredients and processing methods with wholegrain flour yielding higher nutrient density than refined flour (87) fermentation may also influence the level of certain macronutrients and micronutrients in bread (17, 88).

#### Micronutrients vs. anti-nutritional factors

Minerals such as iron, magnesium, zinc, and phosphorus are primarily concentrated in the bran fraction of cereal kernels. Consequently, whole grain flours tend to be richer in these minerals compared to refined flours (89). However, cereals also contain antinutritional compounds such as phytic acid, which has the ability to form insoluble complexes with cationic minerals, thereby reducing their bioaccessibility and their potential absorption by the human body (90). Fortunately, enzymes known as phytases can hydrolyze and mitigate these mineral-phytate complexes. These enzymes are present in the flour and can be also released by yeast and lactic acid bacteria during the fermentation process (91). In the case of sourdough fermentation, the acidic environment optimizes the activity of phytase enzymes, leading to a reduction in phytic acid content. In 1992, Fretzdorff et al. (92) found that the optimal pH range was between pH 4,3–4,6, and 13 years later, Leenhardt et al. (93) showed that a slight decrease of dough pH (5.5) could be sufficient to reduce 70% of phytic acid and make minerals more bioaccessible. This suggests that endogenous phytase could be the main actors of phytic acid reduction, but with an optimal environment offered by fermentation (94, 95).

The enhancement of nutrient bioaccessibility is therefore dependent on the raw material used, in particular the initial phytic acid content in the flour, as well as the endogenous phytases. In this respect, the sourdough fermentation has potential in yielding bread with accessible nutrients in the case of wholegrain or rye bread (89, 96). Ensuring nutrient accessibility through fermentation could therefore not be guaranteed in the case of gluten-free breads, as the source materials differ. Fortification of these products with micronutrients remains a good approach for improving the nutritional density of these products. It is important to note that an increased bioaccessibility of nutrients does not necessarily translate to enhanced bioavailability and physiological benefits, and that clinical studies are still needed to fill the gap between these two concepts.

#### Starch digestibility, fiber content, and glycemic index (GI)

Starch is the primary energy storage in cereal grains and is composed of two main polymeric structures composed of glucose units: amylose which is linear and amylopectin, a highly branched polymer. Starches are classified in terms of digestibility rates: Rapidly Digestible Starches (RDS), Slowly Digestible Starches (SDS), and Resistant Starches (RS) that are not digested in the small intestine and reach the colon during the digestion process (97). RS are considered as dietary fibers (98), and are associated with positive health effects, such as flattening of postprandial glycemic responses (99). This effect is partly obtained thanks to the crystalline organization of starch granules. During breadmaking however, particularly the baking process, the starch granules undergo gelatinization, where the starch molecules absorb water, swell, and lose this crystalline structure. This gelatinization process makes the starch more susceptible to enzymatic hydrolysis by digestive enzymes like α-amylase, which may result in an increased GI (100–102).

Nonetheless, sourdough fermentation has been reported to increase the RS content in specific baking conditions or using selected starters (102, 216). This effect appears more consistent when using whole wheat flour (103). Others reported that using sourdough fermentation of gluten-free flours generated discrepant results on the final RS content or glycemic index of gluten-free bread (43, 104, 105). It seems however that sourdough has a positive influence on starch retrogradation during staling, both on wheat-based (106) or gluten-free bread (107), which may positively affect bread's GI over time.

With the current evidence available, it is not possible to establish that fermentation type could be used *per se* to enhance resistant starch content or glycemic index of gluten-containing or gluten-free breads (108). Still, further investigations related to the combination of consortium of lactic acid bacteria and yeast with specific flours in determined baking conditions could help producing bread with a lower GI. Additionally, studies are warranted to establish whether these breads generate a more favorable glycemic response in a clinical setting.

### Using fermentation for enhancing palatability

Another promising functionality of microorganisms interaction during sourdough fermentation lies in its ability to improve the palatability of food (88, 96). Dietary fiber intake should be increased in the modern western diet to reduce the risk of NCDs, such as obesity, colorectal cancer, coronary heart disease and type 2 diabetes (109). Unfortunately, addition of fiber in breadmaking greatly alters the dough gas retention capacity leading to a bread with a decreased volume, a denser, harder and coarser crumb and bitter taste (110). Salmenkallio-Marttila et al. (111) showed that pre-fermentation of bran with yeast and more effectively, with yeast and lactic acid bacteria improved loaf volume and crumb softness during storage. The addition of wheat sourdough produced in optimized conditions was also effective to improve the overall quality of mixed oat/wheat bread to produce satisfactory bread with increased content in oat β-glucan (112). Also, sourdough with selected lactic acid bacteria and yeast starters consortia was reported to enhance aroma and flavor of 100% whole-oat bread (113).

Bread is mostly made with wheat flour due to the unique viscoelastic properties of its gluten network, enabling the dough to retain gas and rise during proofing and baking. Yet partial substitution of the flour with more climate-resilient crops could help reduce the need for wheat flour and ensure food security worldwide (114). Moreover, the wide variety of flours available to date coming from either cereals, pseudocereals, tubers, or legumes opens new potential for bread with improved nutritional value, particularly when combined with fermentation. Several studies acknowledged the positive impact of alternative flours and fermentation on bread nutritional values, but they also report alterations in bread properties (115–119). Therefore, there is a great need for further improvement of the sourdough fermentation and breadmaking processes to optimize bread organoleptic qualities (114).

Another growing sector with a significant need for gluten substitutes is the formulation of GF bread. GF dough shows lower cohesiveness and elasticity compared to wheat dough. The lack of gluten results in bread with a poor texture and color and characterized by lower specific volume. Shorter shelf life, dry mouth feeling and unsatisfying taste leading to the development of expensive and complex not clean label formulations are some of the problems faced by researchers, bakers, and the food industry to date (120–122).

Several studies investigated the effects of sourdough fermentation on GF bread in the hope of improving its palatability, while reducing the use of additives. Matos and Rosell observed that sourdough fermentation positively influenced several aspects of bread quality, i.e., texture, aroma, nutritional properties, and shelf life but that the results depended on sourdough microbial composition (121). The acidification brought by sourdough fermentation enhanced polysaccharide swelling, increased GF dough elasticity, better bread volume and delayed staling in GF bread (120, 123–125). Conversely, Moroni and colleagues showed negative effects of sourdough fermentation on GF buckwheat dough and bread quality (126). Promising results have also been shown by the attempt to produce exopolysaccharides (EPS) in GF dough during fermentation, to replace commercial hydrocolloids or gums in mimicking gluten network properties. EPS, synthesized *de novo* by various strains of lactic acid bacteria during growth and fermentation, are long-chain polysaccharides that can exist as capsular or ropy slime layers. These biopolymers play a crucial role in determining the rheological, textural, and stability properties of fermented foods, while also exhibiting potential health-promoting benefits. Lynch et al. (127) concluded that EPS produced in GF dough by LAB have the potential to enhance loaf volume, shelf-life and staling rate of GF bread. Once again this requires further investigation to properly identify safe and high yield EPS-producing LAB strains, optimal flours to be used as substrates and fine-tuned fermentation and breadmaking processes (128).

Lowering salt levels is a key challenge in the baking industry. Indeed, salt is one of the four basic ingredients of bread and has different roles: dough strengthening, fermentation control, flavor enhancement and in the preservation of the bread (129). But sodium intake is also a risk factor for cardiovascular diseases and bread can be a major contributor to this intake (3). This is why salt reduction is at the heart of certain food policies and is becoming mandatory in several regions around the world (130). Sourdough fermentation is one of the existing solutions to reduce salt content (129) without affecting the bread characteristics. Also, the presence of acetic and lactic acids helps to enhance the flavor, impacts the texture and the dough rheology and bread preservation in salt-reduced breads (131, 132).

### Using fermentation for reducing specific components

#### Gluten

In recent years, a range of *in vitro* studies investigated whether lactic acid bacteria strains isolated from sourdough, alone or in combination, could hydrolyze protein fractions of gluten, thus reducing immunogenicity.

Most of these studies consisted in the selection of bacterial strains with specific proteolysis abilities (i.e., producing a specific pattern of peptidases), and assessed this effect during sourdough fermentation. In several studies, these strains were able to effectively degrade the 33-mer epitope, responsible for immune reaction in celiac individuals, or other fragments of gliadin (133–137). However, results are conflicting, as other authors reported only partial degradation of α- and γ-gliadins (138).

Interestingly, one research team highlighted an increased content in 33-mer epitope following *in vitro* digestion of commercial wheat sourdough breads compared to yeast bread, resulting in an increased immunoreactivity. However, this was not observed for wheat ciabatta rolls, whatever their sourdough content or fermentation time, showing an average a 46% reduction in this peptide level. Even so, testing these rolls using human sera of three immunosensitive patients provided very different immune responses. The authors noted that the proteolysis occurring during sourdough fermentation of the commercial breads may have released epitopes that remained in the matrix without further degradation (211). In another study, a type-I sourdough wheat fermentation showed no proteolysis of gliadins, and even increased antigenicity compared to a white wheat bread, possibly due to a partial hydrolysis occurring during fermentation (212). This suggests that native sourdough fermentation is ineffective in reducing immunogenicity of gluten.

The role of lactic acid bacteria *per se* in gluten degradation is somewhat controversial. In several studies, no differences between sourdough and chemically acidified dough could be highlighted on gluten degradation (139, 140). In other studies however, different patterns of gliadins or glutenins degradation were observed between sourdough fermented and chemically-acidified doughs (138, 141). Gerez et al. (142) reported that wheat dough with cell-free extracts could degrade gliadins more effectively than wheat dough with LAB. These results indicate that a significant part of the effect may be due to the activation of indigenous cereal enzymes together with LAB peptidases that are activated by the decreasing pH. Regarding yeasts, Méndez et al. (143) highlighted that the *Wickerhamomyces anomalus* strains could reduce the immunogenic α-gliadins by up to 78% in bread.

One question arising when considering the gluten proteolysis capacity of the sourdough process is whether the decrease could be sufficient or standardized to produce bread suitable for celiac disease patients. In this respect, Engström et al. raised concerns, as in their *in vitro* study, sourdough fermentation was unable to effectively prevent the binding of transglutaminase 2 (playing a crucial role in the initiation of celiac disease) to gluten peptides. The authors even noticed that prolonged fermentation may increase binding sites on α2-gliadin, and concluded that lactic acid fermentation may not be an adequate strategy for producing celiac safe products (144).

Noting the current uncertainties, it appears that the use of sourdough fermentation for gluten degradation would be more suitable for preventing the risk of cross-contamination of gluten-free products than for producing gluten-free bread (145).

Nonetheless, other strategies were developed for more extensive gluten degradation. Notably, Rizzello et al. used a specific set of lactic acid bacteria together with fungal proteases (from *Aspergillus oryzae*, and *Aspergillus niger*). They demonstrated that using this combination in wheat sourdough yield a final gluten level of 12 ppm, less than the 20 ppm regulatory threshold for labeling gluten-free products. Also, hydrolysis of the 33-mer gliadin epitope was 70% after 6h, and complete after 18h of fermentation (146). Similar findings were reported later by another study in durum wheat semolina fermented with the same association (147).

It should be noted that most of the findings mentioned above were obtained from non-clinical studies. Still, baked goods made using similar processes were reported to be suitable in celiac disease patients in clinical settings (213–215). However, further clinical studies are still warranted to allow extrapolation to consumers. Also, while this approach appears promising for making gluten-free products, its use for industrial application appears impractical. One key challenge is the ability to produce bread with satisfactory textural and organoleptic properties, as disrupting the gluten network impairs its structuring properties, and requires additives for compensating the loss (144, 145). Nonetheless, one study reported obtaining breads with acceptable sensory and nutritional characteristics in such conditions (148).

#### FODMAPs

FODMAPs are found in significant amounts in foods such as garlic, onion, artichoke or leek, and to some extent in rye products and wheat bran (149). Individuals suffering from IBS ingesting those foods may experience gastrointestinal symptoms such as alterations in bowel habits, abdominal pain or distension, bloating or flatulence (150). A diet low in FODMAPs has been shown effective to reduce gastrointestinal symptoms and quality of life in individuals suffering from IBS (151).

FODMAPs content of breads were reported to range between 0.24 and 3.31 g/100 g dry matter (DM) depending on the cereal, process and fermentation type (152, 153). Main FODMAPs commonly present in wheat breads are fructans but others such as fructose and galacto-oligosaccharides are also present.

A range of studies have demonstrated the ability of selected microorganisms to reduce significantly FODMAPs content in bread products (154). Some strategies consisted in the selection of yeasts, based on their invertase or inulinase activities, showing a decrease of 90% of fructan content with *Kluyveromyces marxianus* (155), or a reduction in concentration to 0.3% dry matter using a subset of *Saccharomyces cerevisiae* strains (156). Other studies demonstrated extensive fructans degradation capabilities of strains such as *Lachancea fermentati* FST 5.1 (157). Of note, baker's yeast has been shown to degrade more than 90% FODMAPs during breadmaking with its native properties (158, 159). In contrast, traditional sourdough fermentation may not always be effective in this respect (159), notably as the ability of LAB to produce enzymes able to degrade FODMAPs, such as extracellular or intracellular fructanase, is strain-dependent (154). Nonetheless, sourdough fermentation with these selected LAB caused significant FODMAPs degradation in final bread products (160–162). As an example, sourdough with *Lacticaseibacillus paracasei R3*, or *Pediococcus pentosaceus* RYE106 could reduce fructans by 73% in whole-wheat bread (163). Production of bread with low-FODMAPs content using processes that are readily applicable in baking such as baker's yeast fermentation may be helpful, as consuming low-FODMAPs bread was shown to alleviate symptoms of IBS in clinical settings (217, 218).

Still, additional clinical studies are necessary to standardize the processes and ferments used, and to confirm the potential physiological benefits for individuals suffering from IBS.

#### α-Amylase-Trypsin Inhibitors (ATIs)

ATIs are a group of low-molecular proteins that are highly resistant to gastrointestinal proteases and can be found in the endosperm of plant seeds, where they act as natural pesticides. Although the role of ATIs in NCGS remains uncertain, they have been proposed as molecules with the potential to activate the innate immune system, potentially provoking intestinal inflammation (24).

Several studies focused on the potential of sourdough fermentation with specific strains to degrade ATIs. Fraberger et al. (164) identified these abilities for different strains, such as *P. pentosaceus* Pp3, *Lo. coryniformis* Lco4, *Lac. paracasei* Lpa4. Huang et al. (165) showed that, compared to unfermented wheat, sourdough fermentation of wheat with selected bacteria and yeast lowered the pro-inflammatory bioactivity of ATIs. Similarly, Reale et al. (138) reported degradation of these compounds in a wheat sourdough fermentation model. In another study, type-I sourdough fermentation led to a significant reduction in ATIs levels, with up to 41% reduction observed after 12 h of fermentation (18). While yeast fermentation may not significantly reduce their total quantity, it can modify the allergenic epitopes of ATIs, potentially decreasing their allergenicity in some cases, but also exposing or generating new allergenic epitopes in others, leading to a more complex impact on ATIs allergenicity compared to fermentation with certain *Fructolactobacilli* strains (166).

Of note, while reduction of ATI in bread can be achieved through fermentation, this reduction is not always associated with symptoms alleviation in individuals with gastrointestinal disorders/sensitivity (167). Overall, the current evidence regarding the role of ATIs in NCGS is still insufficient, and further clinical investigations are needed before making any dietary recommendation (13).

### Future perspectives related to compounds generated by fermentation and health

Noting the elements presented above, fermentation in breadmaking may be a useful process from a nutrition perspective, as it has potential in reducing anti-nutritional factors or triggering components, and altering the accessibility of nutrients. Further, fermentation is also known to generate metabolites with a positive effect on human health (Table 2).

**Table 2:** Future of fermentation.

Category	Compounds/elements	Findings	Challenges
Use of alternative ingredients	Prefermented bran with yeast and LAB	- Dietary fiber intake help to reduce the risk of non communicable diseases (109) - Fermentation improved loaf volume and crumb softness during storage (111)	- Addition of brans alters the dough gas retention capacity (decreased volume,) and can give a bitter taste (110)
	Flour made from climate-resilient crops (CRCs)	- Help reduce the need for wheat flour and ensure food security worldwide (114)	- Alterations in bread properties (115–119, 148) - Need for optimization of the fermentation and breadmaking processes to optimize organoleptic qualities (114)
Possible bioactives compounds	Production of EPS during sourdough fermentation	- Potential prebiotic effects in bread in preclinical studies (104, 182–186)	- Requires further investigation to identify safe EPS-producing LAB strains, optimal flours as substrates and fine-tuned fermentation and breadmaking processes (161) - Need for clinical trials to confirm the positive effects of EPS in bread on human health
	SCFA (butyrate, propionate, acetate) producing LAB	- Contribute to maintain a healthy colon and protection of colorectal carcinogenesis - Potential beneficial effects on depression, autism, anxiety/stress (187) - SCFA implicated in autoimmune, allergic and metabolic diseases (188)	- Translating effects from animal to human studies limited by physiological and dietary differences and by the challenge of delivering enough SCFA to the target sites for observable effects (188)
	γ-aminobutyric acid (GABA) producing LAB (165, 168)	- Physiological functions in humans: diuretic, anti-depressive, antioxidant and hypotensive (193) - Food rich in GABA could help in the regulation of sleeplessness, autonomic disorders, and depression (194–196)	Further research necessary to identify: - Safe strains, alone or in consortium, with high GABA yield - Fermentation parameters for optimal GABA production (197)
	EpiCor, mix of bioactive molecules produced *in vitro* by a *S. cerevisiae*	- Anti-inflammatory effect (199–201) - Antioxidant activity and biofilm inhibition capacity (202), antitoxin capacity with an impact in vivo on intestinal integrity (203)	- The same metabolites must be produced in dough and their effect confirmed after bread consumption in pre-clinical or clinical trials
Synthetic microbial communities	*Lactobacillus reuteri* LTH5448, *Weissella cibaria* 10M and *S. cerevisiae* in Sorghum Sourdoughs	- Potential to enhance loaf volume, shelf-life and staling rate of GF bread (127)	
	- *L. plantarum* maize sourdough + Baker's yeast (124) - *L. brevis* and/or *L. paralimentarius* pearl millet sourdough (125)	- The acidification brought by sourdough fermentation enhanced polysaccharide swelling, increased GF dough elasticity, better bread volume and delayed staling in GF bread (120, 123–125)	- Negative effects of sourdough fermentation on gluten free buckwheat dough and bread quality (126)

Significant research efforts highlighted the major involvement of the microbiota (sometimes called microbial flora) in maintaining its host health. Indeed, the beneficial association of “lactic acid producing” microorganisms with human was suggested more than a century ago by Döderlein for vaginal bacteria (168). Numerous studies on LAB followed, mainly focusing on Gastrointestinal Tract (GIT). They highlighted that an unbalanced microbiota is associated to a number of Non-Communicable Diseases (NCDs) (169–173).

Diet is one of the main factors contributing to the composition and diversity of the human intestinal microbiota (174). Among the dietary strategies that have gained increasing attention for these effects are probiotics, prebiotics and postbiotics. Probiotics are “*live microorganisms that, when administered in adequate amounts, confer a health benefit on the host*.” (175). A prebiotic is a “*substrate that is selectively utilized by host microorganisms conferring a health benefit*” (176) and postbiotics are defined as “*preparation of inanimate microorganisms and/or their components that confers a health benefit on the host*.” (177).

In fermented bread, probiotics cannot be found, as the viability of microorganisms is lost during the baking process. However, bread may be an interesting source of prebiotics, especially when made with wholemeal flour, as prebiotics are typically non-digestible fiber compounds that pass undigested through the upper part of the gastrointestinal tract, and help growth or activity of advantageous bacteria in the lower GIT by acting as substrates for them (178).

On the other hand, bread, as a fermented food, could be an interesting source of postbiotics. These include bacteriocins, cell wall components, enzymes, peptides, EPS, organic acids, peptidoglycan, short-chain fatty acids (SCFA), vitamins, and other soluble bioactive compounds essential for sustaining human and animal health (179, 180). Postbiotics can influence biological processes by mechanisms including gut microbiota modification, immunomodulation, and anti-inflammatory and antioxidant actions (181). Human clinical trials showed their potential in alleviating symptoms in individuals with gastrointestinal perturbations, reducing stress and anxiety, or promoting anti-inflammatory response (177, 181). While bread could serve as an effective delivery matrix for these preparations, significant challenges remain in validating specific health benefits for particular recipes, processes, and populations, as the exacts mechanisms of postbiotics activities are not yet fully elucidated.

Preclinical studies have demonstrated the prebiotic potential of EPS produced during sourdough fermentation (182–186). However, clinical investigations are necessary to confirm the potential positive effects of EPS in bread on human health.

Butyrate, propionate and acetate are short chain fatty acids (SCFA), important for maintaining a healthy colon and are considered as protective in colorectal carcinogenesis. Many LAB can produce SCFA, with potential beneficial effects on depression, autism, anxiety, and stress as well (187). SCFA are implicated in many autoimmune, allergic and metabolic diseases. However, translating effects of SCFA from animal to human studies is limited by physiological and dietary differences and by the challenge of delivering enough SCFA to the target sites for observable effects (188).

A great deal of interest has also been brought to the production of γ-aminobutyric acid (GABA) by LAB during sourdough fermentation (115, 189–191). GABA is a non-protein amino acid, which acts as the major inhibitory neurotransmitter of the central nervous system (192). It has several physiological functions in humans, such as diuretic, anti-depressive, antioxidant and hypotensive (193). GABA is abundant in fermented foods such as kimchi, cheese and fermented milk products and previous studies showed that food rich in GABA could help in the regulation of sleeplessness, autonomic disorders, and depression (194–196). Further research is nevertheless necessary to identify safe strains, alone or in consortium, with high GABA yield and identify fermentation parameters for optimal GABA production (197). Finally, the actual absorption of these compounds during human digestion and the translation of it to observable physiological effects remain to be determined in clinical studies (113).

Although the focus on potential benefit in breadmaking fermentation is mainly on LAB, the baker's yeast, *S. cerevisiae*, also displayed the capability to produce metabolites and components that may also have health effect, such as vitamins, polyphenols, sterols, and phospholipids (198). *In vitro, S. cerevisiae* produced EpiCor, a complex mixture of bioactive molecules, with an anti-inflammatory immunogen effect (199–201). The yeast metabolites also displayed antioxidant activity and biofilm inhibition capacity (202), antitoxin capacity with an impact *in vivo* on intestinal integrity (203). The same metabolites now have to be produced in dough and their effect confirmed after bread consumption in pre-clinical or clinical trials. In a recent review, Franco underlined the importance of extending research on yeast postbiotics as they remain minor compared to LAB, despite showing great potential (204).

## Conclusion

There is an increasing prevalence of reported gastrointestinal symptoms and sensitivities related to gluten or wheat in the general population, outside of the well-characterized celiac disease and wheat allergy. NCGS is a term used to encompass these sensitivities, though this condition is poorly characterized and associated with a variety of factors and potential causative agents, either physiological or psychological.

Fermentation has been a viable strategy over years to improve nutritional quality, gastrointestinal tolerability or carbohydrate digestibility of foods. The fermentation process enables modifications of proteins, carbohydrates, fibers, and bioactive compounds, and recent advances in understanding microorganisms in the context of fermentation allows targeting components that generate gastrointestinal issues, thus giving the possibility to produce foods suitable for individuals with specific sensitivities. Precise control over the fermentation process, through careful selection of yeast and lactic acid bacteria strains as well as the optimization of process parameters can enable the customization of bread characteristics to meet the needs of different consumer groups, such as the production of lower glycemic index bread suitable for diabetic patients or the use of specific LAB or yeast strains to reduce the content of FODMAPs for individuals with IBS or NCGS.

Nonetheless, gaps in research remain mainly due to the complexity of bread making process, recipes and inter-individual variability. As such, human studies showing the translation of these fermentation-based benefits into a clinical setting are still needed, while the explanatory factors surrounding NCGS and associated conditions remain to be fully understood.

In the meantime, future innovations related to new metabolites produced by fermentation open new perspectives in fermented foods optimization, notably bread, and its benefits on digestive or metabolic health.
